# Individual and seasonal variation in the movement behavior of two tropical nectarivorous birds

**DOI:** 10.1186/s40462-021-00275-5

**Published:** 2021-07-07

**Authors:** Jennifer R. Smetzer, Kristina L. Paxton, Eben H. Paxton

**Affiliations:** 1 Hawaiʻi Cooperative Studies Unit, University of Hawaiʻi at Hilo, PO Box 44, Hawai‘i National Park, HI 96718 USA; 2 U.S. Geological Survey Pacific Island Ecosystems Research Center, PO Box 44, Hawai‘i National Park, HI 96718 USA

**Keywords:** ʻApapane, Avian malaria, Hawaiian honeycreeper, ʻIʻiwi, Intraspecific variability, Movement strategies, Nectarivore

## Abstract

**Background:**

Movement of animals directly affects individual fitness, yet fine spatial and temporal resolution movement behavior has been studied in relatively few small species, particularly in the tropics. Nectarivorous Hawaiian honeycreepers are believed to be highly mobile throughout the year, but their fine-scale movement patterns remain unknown. The movement behavior of these crucial pollinators has important implications for forest ecology, and for mortality from avian malaria (*Plasmodium relictum*), an introduced disease that does not occur in high-elevation forests where Hawaiian honeycreepers primarily breed.

**Methods:**

We used an automated radio telemetry network to track the movement of two Hawaiian honeycreeper species, the ʻapapane (*Himatione sanguinea*) and ʻiʻiwi (*Drepanis coccinea*). We collected high temporal and spatial resolution data across the annual cycle. We identified movement strategies using a multivariate analysis of movement metrics and assessed seasonal changes in movement behavior.

**Results:**

Both species exhibited multiple movement strategies including sedentary, central place foraging, commuting, and nomadism , and these movement strategies occurred simultaneously across the population. We observed a high degree of intraspecific variability at the individual and population level. The timing of the movement strategies corresponded well with regional bloom patterns of ‘ōhi‘a (*Metrosideros polymorpha*) the primary nectar source for the focal species. Birds made long-distance flights, including multi-day forays outside the tracking array, but exhibited a high degree of fidelity to a core use area, even in the non-breeding period. Both species visited elevations where avian malaria can occur but exhibited little seasonal change in elevation (< 150 m) and regularly returned to high-elevation roosts at night.

**Conclusions:**

This study demonstrates the power of automated telemetry to study complex and fine-scale movement behaviors in rugged tropical environments. Our work reveals a system in which birds can track shifting resources using a diverse set of movement behaviors and can facultatively respond to environmental change. Importantly, fidelity to high-elevation roosting sites minimizes nocturnal exposure to avian malaria for far-ranging individuals and is thus a beneficial behavior that may be under high selection pressure.

**Supplementary Information:**

The online version contains supplementary material available at 10.1186/s40462-021-00275-5.

## Background

Movement of animals over space and time directly affects individual fitness, influences the structure and dynamics of populations and communities, and is a key component of ecology and evolution [1]. Animals move to secure mates, avoid danger, gather social information, and acquire resources that vary over space and time [2, 3]. Movement is also a key mechanism by which animals respond to environmental change over evolutionary time scales [4]. Movement decisions have high fitness consequences and are under strong selection, so study of movement behavior can provide deep ecological and evolutionary insights [5, 6].

Animals have evolved a variety of movement strategies to maximize fitness and resource allocation, with range residency, migration, and nomadism common across vertebrate taxa [7]. Nomadic movement is highly variable in orientation and timing and is a response to nonseasonal, unpredictable environmental variability for which territorial defense or ingrained migratory behavior are less beneficial [8]. In contrast, migratory movements are an adaptation to predictable seasonality in resources, and range residency is most adaptive in environments with stable, evenly distributed, but finite resources [9]. Range residency (i.e. fidelity to a core area) can be sub-classified into finer-scale movement strategies including territoriality, in which movement is confined to a discrete and defended area [10], central place foraging, wherein animals repeatedly return to the same location between foraging trips to provision young [11, 12], and commuting, which can be an extreme case of central place foraging [13], in which animals move between roosting or nesting areas to geographically distinct feeding sites [14–16]. Even typically sedentary animals can embark on occasional long-distance forays to access seasonal resources, gather information, or prospect for foraging and breeding sites [17, 18], indicating flexibility and individual variation within movement strategies.

Though intraspecific variation in movement strategies is widespread, and observed across taxa in both marine and terrestrial environments [7], less than 20% of movement research has evaluated intraspecific variability in movement behavior, or gradients of variation within a movement strategy [2]. Intraspecific variation in movement behavior can occur between individuals in a population [17, 19, 20], across the course of an animal’s lifetime [5, 21], between populations [22], and at all these scales within a single species [23]. Intraspecific movement variability is most ubiquitous in unpredictable environments, in which individuals must learn about the spatial and temporal distribution of resources and in species that can flexibly change strategies to maximize fitness throughout their life as they gain experience and social status [23, 24]. Plasticity in movement behavior also has important implications for how well animals can behaviorally respond, and ultimately adapt to environmental change [2].

With a rich diversity of habitats and species, the tropics present a particularly valuable system for understanding the ecology and evolution of movement behavior. However, the majority of ecological research is based on temperate systems, creating a potential bias in our understanding of ecological systems, especially for birds [25]. Since tropical birds face less seasonality, greater biotic selection pressure, and exhibit divergent life history traits relative to their temperate counterparts [26], the ecological and evolutionary context of avian movement behavior likely differs markedly in tropical and temperate systems. Tropical birds also exhibit a diversity of movement strategies [27], and thus offer a rich but under-utilized opportunity to investigate the ecology and evolution of movement behavior [28].

Nectarivorous birds exemplify the diversity of movement behavior in the tropics, due to exceptional temporal and spatial variability in both food resources and competition [29]. Nectar is a temporally and spatially unpredictable resource with temperature, season, elevation, soil substate, and genetic varieties all influencing the variability and intensity of bloom [30, 31]. Reflecting this, tropical nectarivores range from sedentary, territorial species that rely on synchronous smaller nectar resources, to highly nomadic species that track asynchronous, superabundant blooms [32, 33]. Altitudinal migration is also common in tropical nectarivores [6, 34]. Though commuting behavior has yet to be documented in tropical nectarivores, the large distances that some species appear to move to track spatially irregular bloom patterns indicates that commuting behavior may occur. Intraspecific variation in movement behavior is also well documented; at the species level, nectarivores can include territorial, nomadic, and migratory individuals [35]. At the individual level, nectarivores can also switch from residential to highly mobile throughout their lives in response to nectar availability [36, 37], and from territoriality to central place foraging, with multiple strategies used concurrently by one bird [38].

Though modern tracking technologies have begun to offer unprecedented insight into the movement ecology of tropical birds [32, 39], tracking studies documenting movement behavior are still under-represented in the tropics, especially for small-bodied species [27]. To address this gap, we used an automated radio telemetry network to quantify the seasonal movement behavior and elevational patterns of two nectarivorous Hawaiian honeycreepers, ʻapapane (*Himatione sanguinea*) and ʻiʻiwi (*Drepanis coccinea*). The Hawaiian Islands exemplify a simplified tropical system, with an endemic avian community that has low taxonomic diversity but a diversity of foraging guilds, and thus offer a unique study system for investigating the ecology and evolution of movement behaviors. As with other small-bodied tropical nectarivores, most information on the movement behavior of Hawaiian species is based on point count data, mist-net capture, stable isotope data, or visual observations, rather than individual-based telemetry data. The use of a landscape-level tracking network with fine spatial and temporal resolution thus allows us to characterize movement behaviors in ways not possible with other technologies.

The primary goals of our study were to identify movement strategies in our focal species, quantify individual variation in movement behavior, and assess whether seasonal changes in movement behavior corresponded with resource phenology. ʻApapane and ʻiʻiwi both rely on nectar from ‘ōhi‘a (*Metrosideros polymorpha*), a dominant tree species found throughout Hawaiian forests from tree line (2500 m) to sea level [40], though ʻiʻiwi also forage on nectar from a variety of other understory plants [41]. ‘Ōhi‘a is a particularly unpredictable nectar source as it not tightly regulated by environmental cues [42, 43]. Individual trees produce low-level blooms year-round, but also can also erupt in super-abundant blooms that are locally asynchronous across sites, elevations, and genetic varieties [30, 31]. Three years of previous work at our study site documented 1) consistent bloom of ‘ōhi‘a at high elevations from late-winter to spring, and 2) that once the localized blooms declined, ʻiʻiwi regularly moved 10+ km to forage at an intense superabundant bloom that occurred May-Aug. at a lower elevation area to the north-northeast [44, 45]. Though we did not collect data on nectar resources in this study, we tracked our focal species at the same study site so we could evaluate the observed movement patterns in the context of previously documented patterns of resource phenology at the study site.

A secondary objective of this work was to better understand how movement strategies influence exposure to avian malaria (*Plasmodium relictum*), an introduced parasite responsible for significant mortality in native Hawaiian birds [46–48]. Naturalists have long noted ʻapapane and ʻiʻiwi making long-distance flights, presumably to track the shifting distribution of blooms [49, 50]. Though there is quantitative evidence of seasonal elevation movement for both species [44, 51], it is unclear whether long-distance flights constitute nomadic movements, elevational migrations, central place foraging, or commuting. This distinction has important implications because sustained elevational movements can increase exposure to avian malaria. Year-round disease transmission of this disease is currently limited by temperature to areas below ~ 1475 m, though transmission can occur up to ~ 1715 m during the warmest months of the year, when the primary vector the southern house mosquito (*Culex quinquefasciatus*) can occupy higher elevation forests [52, 53]. Thus, even birds from high-elevation breeding populations can be exposed to avian malaria if they move to low-elevation areas with high disease prevalence [44], particularly if they remain at night when the mosquito vector is active [48]. Different movement strategies may therefore vary in both the benefits they confer in tracking nectar resources and the degree of risks they pose in terms of avian malaria.

## Methods

### Study area and field methods

The study was conducted at Hakalau Forest National Wildlife Refuge (HFNWR; 19°51′N, 155°18′W), a 13,240 ha preserve on the eastern slopes of Mauna Kea, Hawai‘i Island, in one of the largest tracts of forest left in Hawai‘i. The refuge spans an elevation of 1200 m, which produces a gradient of precipitation and temperature with an average annual rainfall of 2500 mm (range 2159–7620 mm) and a mean daily air temperature of 15 °C (range 11–19 °C) [54]. The study occurred in the upper elevations of the forest, a montane wet forest dominated by ‘ōhi‘a, and koa (*Acacia koa*) with a diversity of understory plants. Although the refuge has a long history of disturbance and an understory dominated by exotic pasture grasses, substantial restoration has occurred in the form of ungulate removal, fencing programs [55], and large-scale reforestation of trees and understory plants [56].

We captured birds in mist nets from Jan. 2014-Jun. 2016 at a high-elevation site in the Pua ‘Akala tract (1883 m), and at two lower sites (1619, 1669 m). All sites were within 5 km of each other. We aged and sexed birds when possible based on plumage, skull pneumatization, morphometrics, and breeding characters, and determined breeding status based on cloacal protuberance swelling and brood patch development [57, 58]. We fitted birds with aluminum U.S. Geological Survey bird bands, and VHF radio transmitters from JDJC Corp (Fisher, Illinois, USA; 4–6 week battery life) using a modified leg loop harnesses [59]. The transmitters were < 5% of body mass for all birds.

**Fig. 1:**
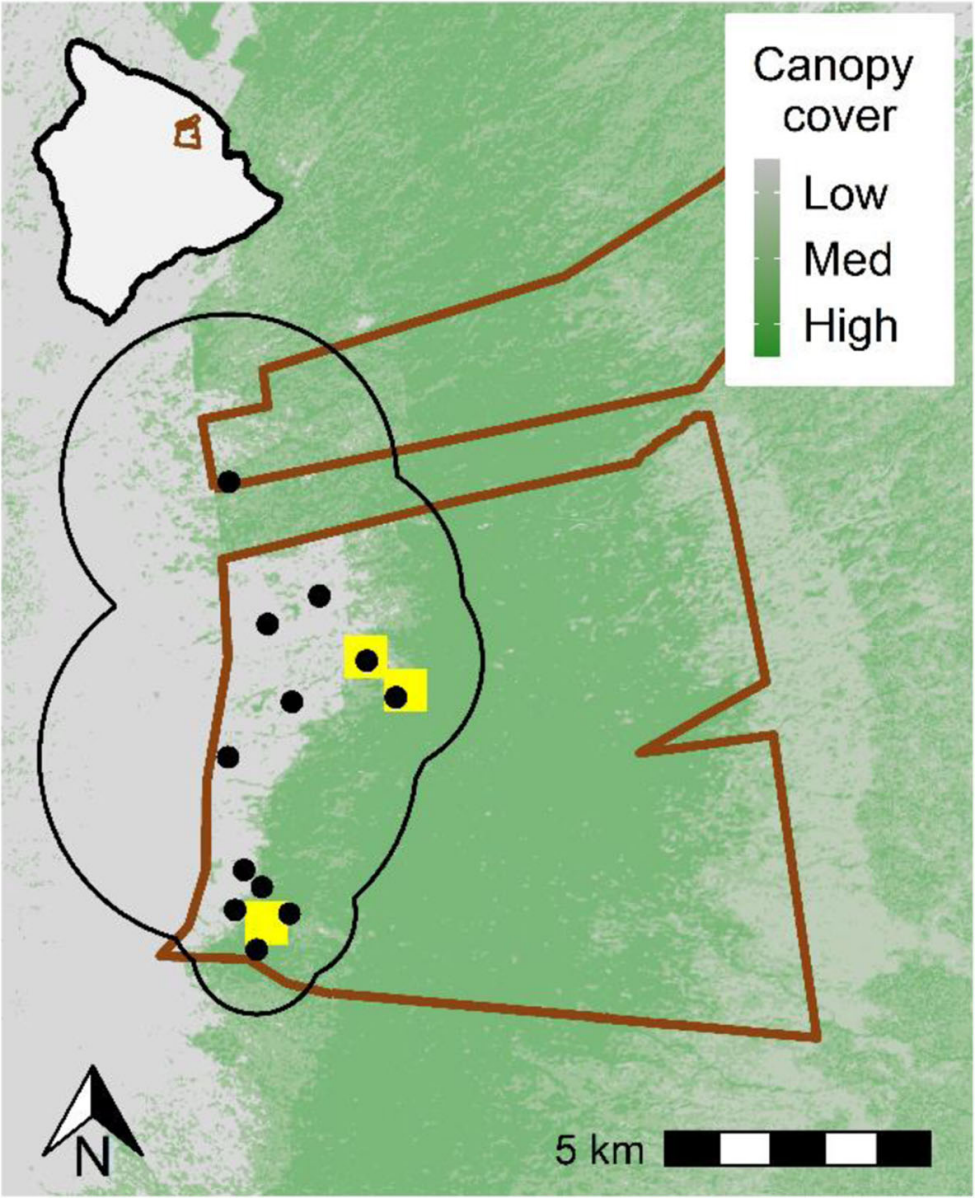
Study area, with Hakalau Forest National Wildlife Refuge boundary in brown, banding sites in yellow, and automated telemetry towers as black points. Birds were detectable within the full interior of the detection area, which is depicted with a black line. Elevation contours in the study area run approximately north-south, with lower elevations to the east and highest elevations to the west. The location of the study site on the Island of Hawai’i is shown in the inset

We tracked the radio tagged birds from Jan. 2014 to Jun. 2016 using a network of 12 automated VHF radio telemetry stations (ARTS; Fig. 1). Each ARTS station consisted of six fixed-Yagi antennas oriented 60° apart on 6–10 m masts, and an automated Sparrow System receiver (JDJC Corp) that listened for radio signals on each frequency every 1–2 min. The receivers were able to detect tags above and below the canopy. We located the ARTS stations in open forest at upper elevations (1636–2005 m), with some above tree line on prominent hills overlooking the forest and others below the canopy near the banding sites to increase detection coverage. Detection ranges were 5.6 and 2.7 km for above canopy and below canopy towers, respectively, and spanned an elevational range of 1543 to 2287 m. We recorded peak signal strength for each transmitter on each antenna, and used the relative signal strength between adjacent antennas to calculate bearings [60].

### Telemetry data

All analyses were conducted in the R software environment version 3.6.3 [61]. To filter for false positives, we excluded signals below the background radiation threshold (− 125 dB), and any detections for which the recorded pulse width and interval were not within 5 and 100 ms respectively of each transmitter’s pulse specifications. We used the telemetr package [62] to estimate locations via maximum likelihood using the signal strength and bearing of synchronized detections [63]. We used training tags at known locations to model signal strength as a function of distance to the receiver and used this relationship to predict the location of tags during single detections. To exclude improbable points we removed location estimates > 10 km from the receivers, and applied a 15 m/s speed filter using the trip package [64, 65]. For all simultaneous detections of training tags, we used simple linear regression to relate localization accuracy to potential predictors of localization error, including distance to tower, median and maximum signal strength value during simultaneous detection, and the total standard error in x and y of localization (total SE) as estimated by the telemetr package. We used the best resulting model – which was determined by Akaike information criteria (AIC) [66] to include total SE – to estimate error for bi or tri-angulated bird locations. We calculated mean localization error above and below − 105 dB for single detections of training tags as there was a clear threshold at this value and used these mean localization error values to estimate error for bird locations based on single detections. The localization routine for training tags resulted in an error of 101 ± 78 m for estimates from ≥3 detections, 157 ± 89 m for estimates from two detections, 190 ± 140 m for estimates from single detections ≥ − 105 dB in signal strength, and 370 ± 330 m for single detections < − 105 dB. We excluded location estimates with > 400 m error and resampled each bird’s track to include only 10% of location estimates from single-detection events, to ensure a similar detection profile across individuals.

To improve the spatial resolution of the data, we used time-series kriging [67], in which the autocorrelation structure of a continuous-time stochastic process (CTSP) model is leveraged to inform localization. Unlike other approaches requiring a priori model choice (e.g. correlated random walk model), time-series kriging in the ctmm package [68] allows users to test models with a range of multi-scale autocorrelation structures [67]. We inspected variograms for each bird, as an asymptote indicates range-residency [69]. We fit models incorporating telemetry location error, and selected among all range-resident CTSP models using AIC corrected for small sample size (AICc [66, 69];). We tested models and ran time series kriging for each bird’s nocturnal and diurnal points separately, as these diurnal species exhibited markedly different movement patterns during diurnal and nocturnal periods. The data were highly regular in sampling interval, with over 99% of detections separated by < 1.5 h, and 90% of temporal gaps missing only one location; however, there were occasional gaps of several days over which we were unable to interpolate locations with reasonable accuracy using spatiotemporal kriging. We thus performed kriging only over the observed data (median sampling interval of 2 min), in the interest of maintaining high spatial resolution location data. The dataset supporting the conclusions of this article is available in the ScienceBase repository at Paxton et al. [70].

### Home range estimates

We used the best diurnal CTSP model for each bird to generate autocorrelated kernel density home range estimates (AKDE) [69]. AKDE estimation uses the CTSP model to calculate an optimal smoothing bandwidth that accounts for autocorrelation [71, 72]. The ctmm package facilitates robust comparison of AKDE home ranges between individuals with different sample sizes, movement patterns, sampling intervals, and telemetry error [73–75].

### Movement strategies

We followed the approach of Abrahms et al. [7] for identifying movement strategies, which involved calculating a suite of movement metrics, conducting a cluster analysis to identify movement strategies, and performing a principle components analysis on the movement metrics to aid with interpretation of the clusters. We developed a suite of metrics widely used to characterize movement behavior [7, 76]. All metrics were calculated over the full tracking duration for each bird. Two metrics, *maximum net squared displacement* (max NSD) and *standard distance deviation* (SDD) characterize the scale of space use, while two others, *daily change in cumulative area* (Δ area) and *standard deviation in daily net squared displacement* (SD daily NSD) describe daily variability in movement behavior. Max NSD is the maximum squared Euclidean distance between any point and the first point in a track and quantifies long-distance movements. SDD is the average distance of all diurnal points in a track from their center. SDD was correlated with AKDE home range area (*r* = 0.77) and served as a proxy since we could not estimate home ranges for all birds. To calculate Δ area we made a minimum convex polygon (MCP) for every day with ≥15 points. We generated a polygon that represented cumulative space use since the beginning of the tracking duration for each bird, over each tracking day, by merging the MCPS for each day with those of all previous tracking days. We used these polygons to calculate the mean change in cumulative area over time by bird. This metric quantifies daily change in space use with larger values indicating more change [21]. Though central place foraging and commuting behavior can both occur across a range of spatial scales, sedentary/territorial birds should exhibit small values for the above metric, while nomads should exhibit large values [7, 21].

We also calculated three recursion metrics useful for characterizing movement [7, 77]. Over each bird’s full tracking period we calculated 1) the total number of *revisits* within a defined distance radius of locations, 2) *residence time*, the total time spent inside the radius, and 3) *return time*, the time between revisits [78]. Since the tracking duration and sample size were different for each bird, we expressed the number of revisits as a ratio (e.g., a revisitation rate) by dividing the number of revisits by the sample size for each individual. We also relativized the residence time and return time for each bird by dividing these by the tracking duration. Though some researchers have regularized location data before calculating recursion statistics, we did not do so because 99% of the temporal gaps in the data were < 90 mins, and 90% of the gaps were missing only one point. Furthermore, recursion statistics are robust to some irregular sampling or temporal gaps, and analyses have been successfully performed for species exhibiting sampling irregularities consistent with those observed in our study [78].

We used a radius of 150 m for calculating recursion statistics, as variance in residence time peaked at this radius for most birds, indicating a characteristic scale of foraging [77]. Furthermore, 150 m was between the mean error of the kriged locations (33 m) and the median step length (160 m) [78]. Unlike animals making random area restricted searches, central place foraging nectarivores tend to exhibit frequent revisits to specific floral resources with revisits timed to allow nectar reserves to renew [38, 79]. We thus only tallied revisits separated by ≥12 h, in the interest of 1) specifically characterizing locations revisited across multiple days, rather than ones revisited multiple times within a given day, and 2) comparing our results to previous work [7]. Moreover, the 12 h threshold was markedly greater than the temporal scale of most temporal irregularities in the data, thus drastically minimizing any influence that temporal data gaps could have on the number of tallied revisits. Central place foragers in this study would therefore exhibit a high revisitation rate, long return times, and short residence times, due to repeated revisitations to the same foraging sites day after day, whereas territorial individuals that patrol a large proportion of locations would show a lower revisitation rate, short return times, and high residence times.

To identify movement strategies, we performed an agglomerative hierarchical cluster analysis using a Euclidean distance matrix of the seven scaled movement metrics and Ward’s minimum-variance linkage algorithm [7]. We used multi-scale bootstrap resampling in the pvclust package [80] to calculate *p*-values for each cluster. We assessed the stability of the clusters by calculating Jaccard similarities between the original clusters and 1000 new solutions generated by randomly omitting birds, and bootstrap resampling [81]. As another measure of robustness we ran a non-hierarchical cluster analysis using a K-medoid algorithm [82], and calculated Jaccard similarities of the resulting solutions.

To help interpret the clusters, we performed a principal components analysis (PCA) on the movement metrics. We also assessed cluster-wise differences in home range size and each clustering metric using pairwise permutation tests in the rcompanion package [83], with a false discovery rate *p*-value adjustment [84]. To help identify if birds exhibited commuting behavior, we also described patterns of movement within a day, as commuting individuals typically make full day excursions to geographically distinct foraging areas before returning in the evening to roosting or nesting sites [15]. To this end, we specifically calculated the number and duration of excursions each bird made per day from a core area centered on its detection centroid, including any excursion > 5 min. We set the core areas equal to the 50% AKDE isopleth of each individual bird, and for birds without AKDE estimates, to the median for its respective group. We used permutation tests to assess if the frequency or duration of excursions differed by cluster. Lastly, for each cluster, we quantified the frequency of “forays,” which are occasional and sporadic exploratory movements longer in distance and duration than typical foraging events [17, 18, 85]. For the purposes of this study, we defined forays as ≥24 h of non-detection in the ARTS network, followed by subsequent redetection.

### Elevational and seasonal patterns

We assessed seasonal and elevational patterns by analyzing data from birds captured and tracked across different seasons, as the transmitters only lasted 4–6 weeks. We extracted the elevations of each detection from a 30 m digital elevation model [86] aggregated to 60 m to match the resolution of the kriged location estimates. We calculated overall elevation range as the difference between the maximum and minimum elevation by bird and calculated a daily elevation range in the same manner for each bird. We used a Welch’s t-test with unequal variances to compare overall elevational range between species, and between birds captured at the high-elevation Pua ‘Akala tract and those captured at the lower elevation sites. We ran mixed effects models in the nlme package [87] with day of year as continuous trigonometric predictors [88] to model seasonal variability in daily elevation range, minimum daily elevation, and median daily NSD. For minimum daily elevation, we modeled species separately, as they showed distinct seasonal rhythms, and only included birds captured at the Pua ‘Akala tract as capture elevation was confounded with season. Species was a covariate for all other response variables. For all mixed-effects models we transformed variables as needed, assumed a 365-day periodicity, included bird as a random effect, used AIC to compare models, considered variables significant at *α* = 0.01 [89] and judged *p-*values ≤0.01 for either trigonometric predictor as evidence of a non-random seasonal pattern. We reported all summary statistics as a mean ± 1 standard deviation unless otherwise noted.

## Results

We tracked 57 birds for an average of 23.9 ± 8.3 days and estimated 4446 ± 5139 locations per bird. The sample was skewed towards adults for ʻapapane (15/19; 79%) and ʻiʻiwi (36/38; 95%) and skewed towards males with 14 male ʻapapane (74%), 28 male ʻiʻiwi (74%), and four ʻiʻiwi of unknown sex (11%). The ARTS network tracked birds over a 4048-ha area, covering elevations between 1543 and 2287 m. Home ranges averaged 160 ± 309 ha for ʻapapane (*n* = 17), and 176 ± 327 ha for ʻiʻiwi (*n* = 33) but were not calculated for seven birds, five due to sample size, and two that were not range resident.

**Fig. 2:**
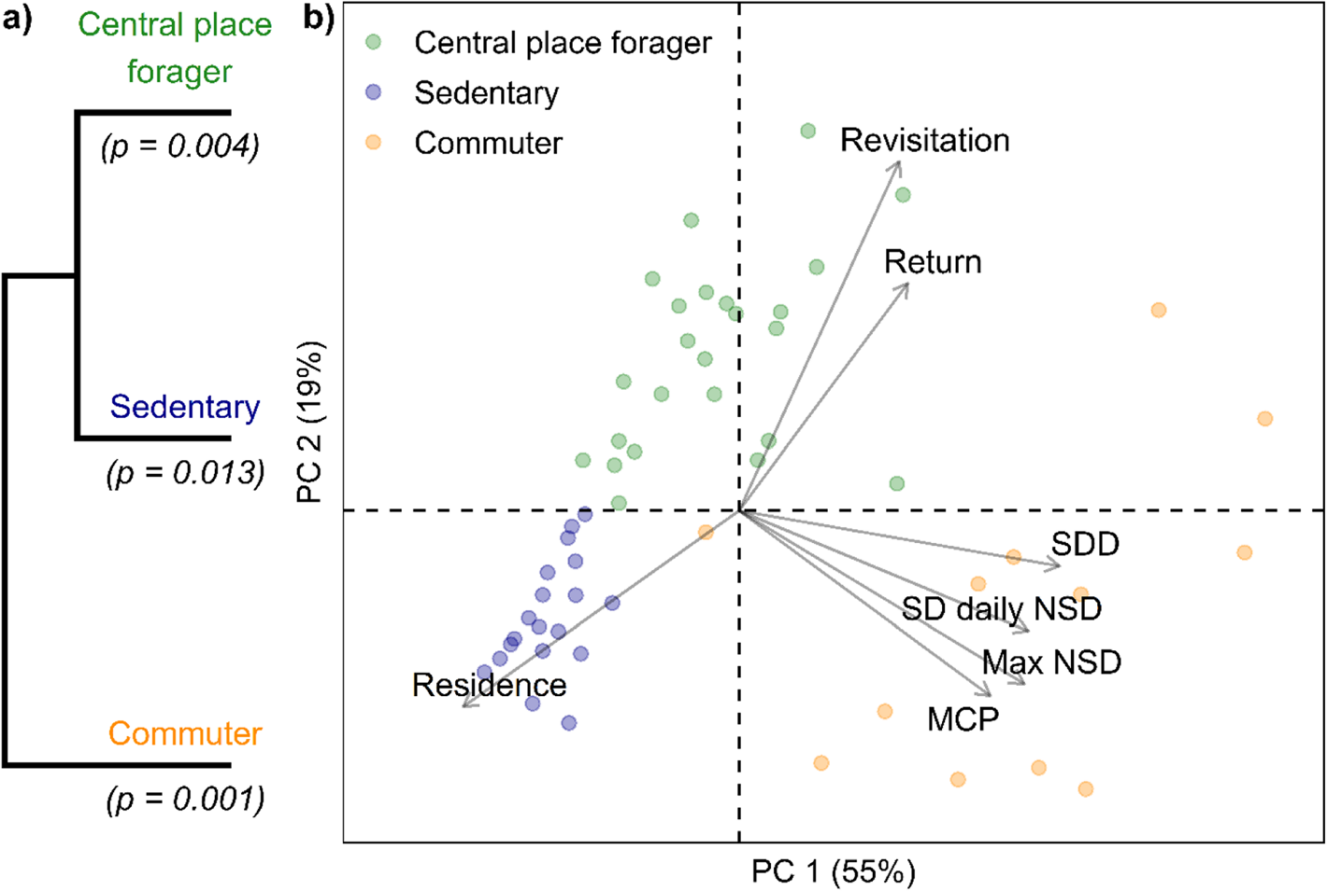
**a** Dendrogram with results of Ward hierarchical cluster analysis, based on movement metrics and bootstrapped *p-*values for each cluster, and **b** Cluster solution on principal components ordination plot of the clustering variables. Points are individual birds, colored by cluster, with ʻapapane (*Himatione sanguinea*) as circles and ʻiʻiwi (*Drepanis coccinea*) as squares. The clustering variables include (Revisitation) rate, or revisits standardized by sample size, (Return) time and (Residence) time standardized by tracking duration, standard deviation of daily net squared displacement (SD daily NSD) maximum net squared displacement (Max NSD), change in daily minimum convex polygon (MCP), and standard distance deviation (SDD) – used as a proxy for home range size. Data are from automated telemetry of ʻapapane and ʻiʻiwi in the Hakalau Forest National Wildlife Refuge from January 2014 to June 2016

The cluster analysis of movement metrics identified three groups deemed significant by multi-scale bootstrap resampling (Fig. 2). The cluster solution was highly stable with Jaccard similarities of 0.91, 0.91, and 0.95 for subsetting randomization, 0.85, 0.89, and 0.89 for bootstrapping, and 0.88, 0.95, and 0.87 for the different clustering methods. Fisher’s exact tests demonstrated that cluster membership was not associated with banding site (*p* = 0.28), species (*p* = 0.64), or age (*p* = 0.81). Three of five birds tagged in separate years changed movement strategies, indicating intra-individual variability. We a-posteriori described the clusters as “sedentary”, “central place forager” (CPF), and “commuter” based on the movement metrics associated with each cluster. The sedentary group contained 42% of the birds (*n* = 24), and the CPF 33% (*n* = 19). The commuter group included 12 birds with behaviors akin to those in the CPF group, but larger in spatial scale, and two non-range-resident birds. A representative example track for each cluster can be seen in the Supplementary Information (Fig. S1).

**Fig. 3:**
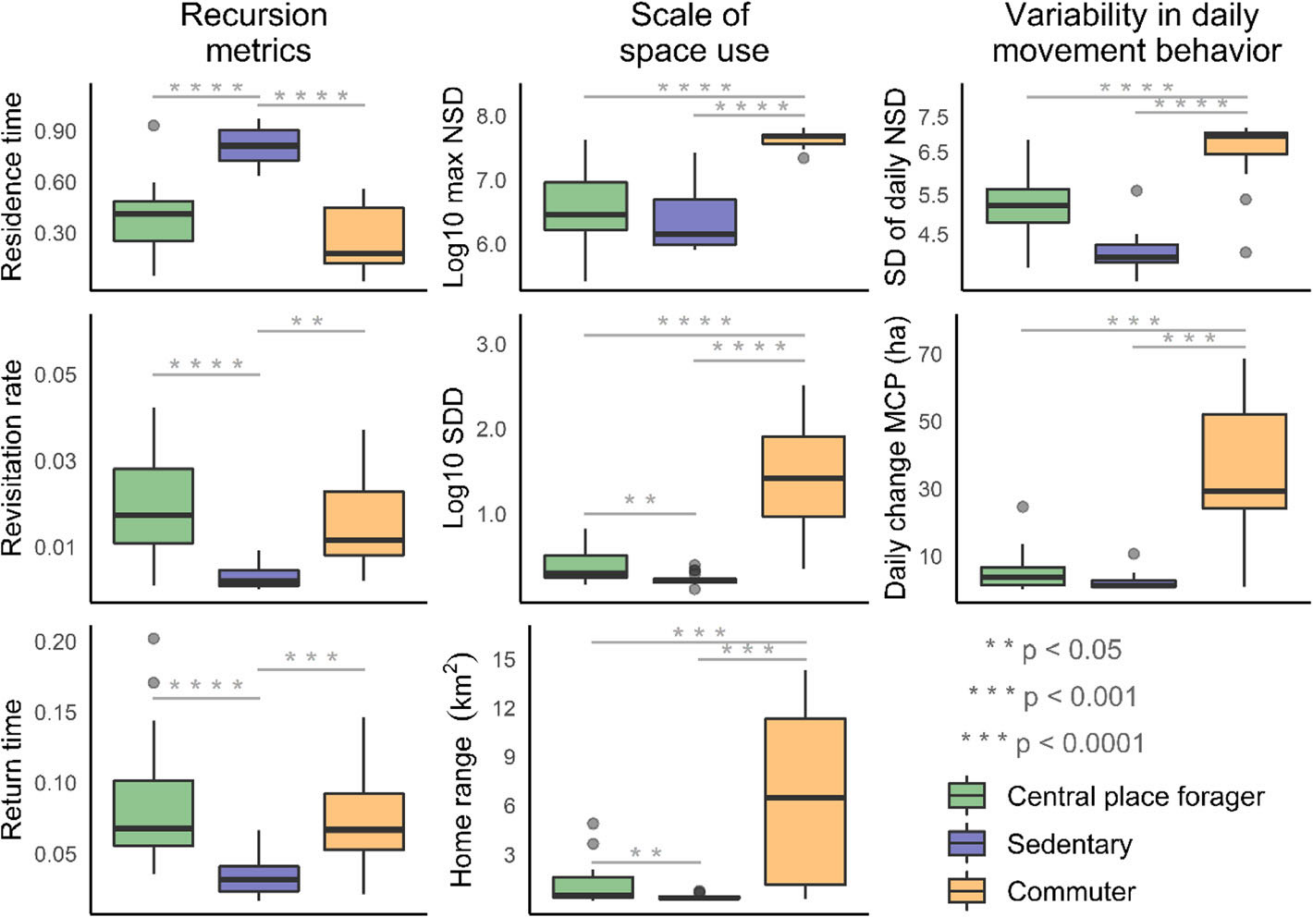
Movement metrics by cluster membership, include (Revisitation) rate, or revisits standardized by sample size, (Return) time and (Residence) time standardized by tracking duration, maximum net squared displacement (Max NSD), home range area, standard distance deviation (SDD) – used as a proxy for home range size, standard deviation of daily net squared displacement (SD of daily NSD), and daily change in minimum convex polygon (MCP). Bars spanning boxplots indicate a significant difference based on pairwise permutation tests, with *p*-values using a false-discovery rate correction. Boxplots show the 25, 50, and 75th quantiles, with whiskers extending from the interquartile range (IQR) to 1.5 × *IQR*, and points depicting values > 1.5 × *IQR*. Data are from automated telemetry of ʻapapane (*Himatione sanguinea*) and ʻiʻiwi (*Drepanis coccinea*) in the Hakalau Forest National Wildlife Refuge from January 2014 to June 2016

The clusters all separated on the first principal component axis (PC1) along a gradient of space use and variability in daily movement behavior, while PC2 largely separated the sedentary cluster from the other two. The loadings indicated that PC1 primarily represented scale of space use (SDD = 0.92; max NSD = 0.82) and variability in daily movement behavior (SD daily NSD = 0.82). The 95% home range estimates also differed significantly between the commuter (665 ± 569 ha), CPF (108 ± 118 ha), and sedentary (36.9 ± 16.4 ha) groups, as did the SDD home range proxy (Fig. 3). Extreme values for variability in daily behavior (Δ area, var. NSD), and max NSD further distinguished the commuter group from the other clusters. The recursion metrics also differentiated the sedentary group from the other clusters along PC2, particularly revisitation rate, the dominant loading on this axis (0.74).

Birds in the sedentary group made multiple short (median 0.3 h; range 0.1–0.5) excursions outside their core areas within a day (median 15 trip/day; range 6–26). However, we recorded few revisits separated by ≥12 h for the sedentary birds indicating that they did not regularly revisit specific locations on the landscape from 1 day to the next. Since the median core area of this group (i.e., 50% AKDE; 4 ha) was smaller than the 7-ha circle used to calculate the recursion metrics, the fact that we recorded few revisits separated by ≥12 h even within the core areas of the sedentary birds demonstrates that they seldom left their primary use areas for extended periods.

**Fig. 4:**
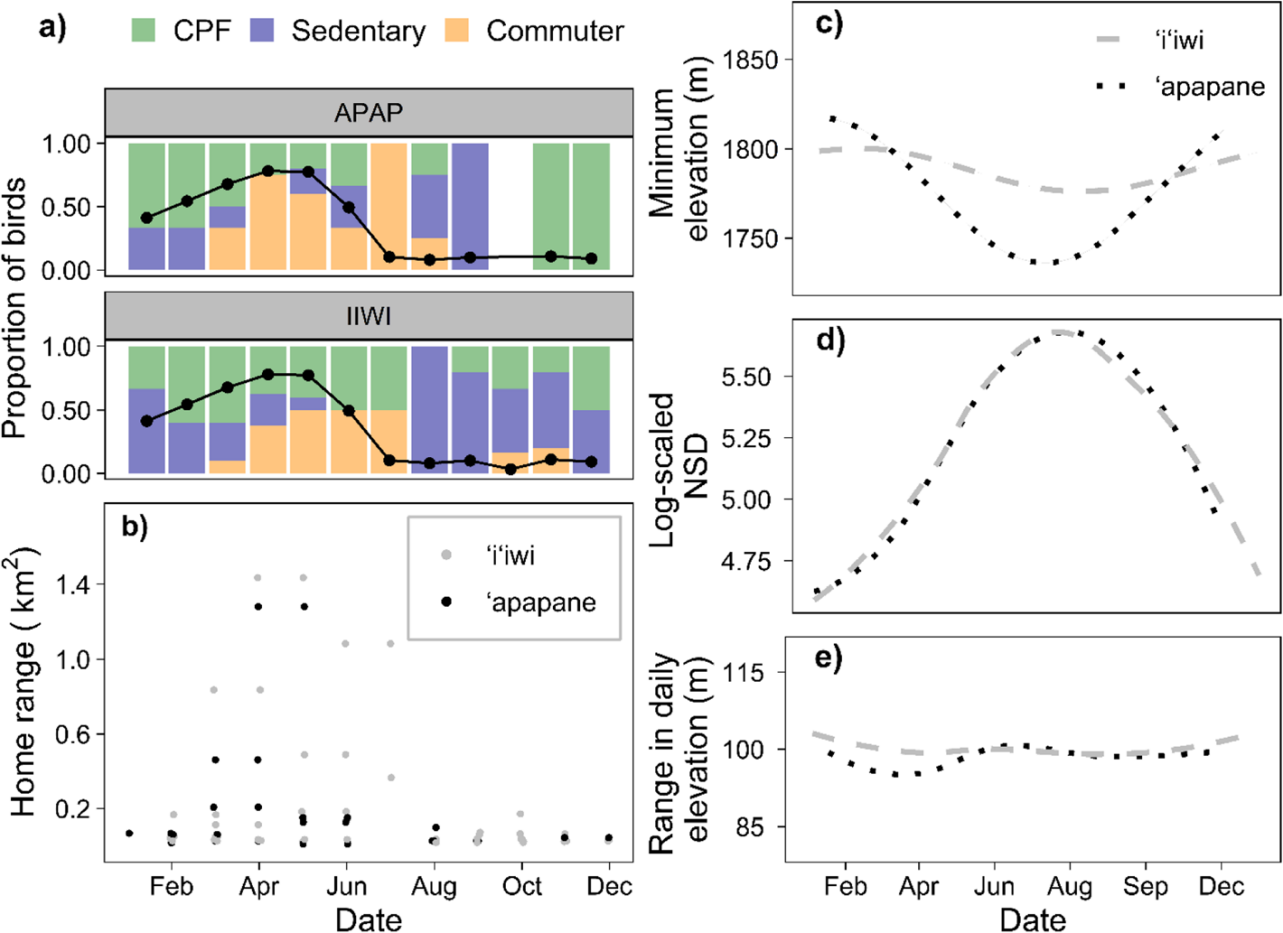
Seasonal patterns for ʻapapane (*Himatione sanguinea*) and ʻiʻiwi (*Drepanis coccinea*) tracked with Figures include **a** proportion of birds in each movement strategy, and in breeding condition (points) by month, **b** home range sizes, with a point for each bird in each month present on the landscape, **c** minimum daily elevation **d** median daily net squared displacement, and **e** range in daily elevation. Data are from automated telemetry in the Hakalau Forest National Wildlife Refuge from January 2014 to June 2016

In contrast, the recursion metrics for birds in the CPF and commuter group reflect large-scale space use as these individuals used core areas that generally exceeded the 7-ha circle used to calculate the recursion metrics (median 50% AKDE = 7, and 68 ha, respectively). Birds in the CPF and commuter group thus regularly travelled outside the 7-ha area used to calculate the recursion metrics, and beyond their core use areas. The CPF birds specifically made between 1 to 20 excursions outside their core area within a day (median 3 trip/day); these excursions were typically short in duration (median 0.7 h; range 0.2–22.4). In contrast, commuters made less frequent (median1 trip/day; range 1–7) but longer daily excursions within a day ranging in duration from 0.3–14.4 h (median 5.8). We recorded a large number of revisits separated by ≥12 h for birds in both the CPF and commuter group (Fig. 4), indicating that they regularly revisited the same specific locations on the landscape from 1 day to the next during excursions. The commuters made fewer excursions per day than the CPF group (*p* = 0.04) and made longer duration excursions than the sedentary group (*p* = 0.001). The sedentary group made more daily excursions than both the CPF (*p* < 0.001), and commuter groups (*p* < 0.001).

Eleven ʻapapane and 17 ʻiʻiwi made ≥24 h forays outside the tracking area with a median of 2.5 forays per bird (range 1–8). Forays were strongly associated with cluster membership (*χ*^*2*^ = 26.2; *df* = 2; *p* < 0.001), but not with species (*χ*^*2*^ = 0.43; *df* = 1; *p* < 0.51). The percentage of birds that made forays outside the tracking area was markedly lower in the sedentary group (1/42; 4%), compared to the CPF (14/19; 74%) or commuter (13/14; 93%). The median duration of forays was 1.7 day for each group, though the maximum duration varied between the sedentary (3 day), CPF (7 day), and commuter group, in which a non-range resident juvenile made a 22-day foray. Forays outside the tracking area occurred in all months, sexes, and age classes.

The behavioral clusters only loosely corresponded to breeding phenology for ʻapapane and ʻiʻiwi (Fig. 4). Based on long-term banding, both species can breed throughout the year, but the peak is Jan.-Jun. Movement strategy was not significantly associated with breeding status at the time of capture (*χ*^*2*^ = 2.4; *df* = 2; *p* = 0.31). Sixty-nine percent of the 39 birds we tagged Jan.-Jun. were in breeding condition at capture, but none of the 19 birds tagged Jul.- Dec had signs of breeding conditions. The CPF group occurred throughout the year but was most common Dec.- Mar., in the early breeding period. The commuter group peaked Apr.- Jul., in the late breeding and early non-breeding period, but one bird in the group was tracked Oct.-Nov. The sedentary behavior occurred in 11 months, but was most common Aug.-Nov., in the late non-breeding period.

ʻApapane and ʻiʻiwi both moved longer distances during the summer months; however, these long-distance movements primarily occurred within elevational bands (Fig. 4). Even though individual ʻapapane and ʻiʻiwi moved up to 7.3 and 7.4 km from their detection centroids, respectively, the mean total elevational range for individuals was only 232 ± 100 m and did not differ between the high (277 ± 106 m) and low-elevation capture sites (225 ± 99 m; *t* = − 1.3; *df* = 9; *p* = 0.22), or species (*t* = 0.80; *df* = 33; *p* = 0.43). Minimum elevations documented for ʻapapane were significantly lower in the summer (*β*_*sin*_ = 5.16; *P* = 0.69; *β*_*cos*_ = 40.28; *p* = 0.001; *R*_*c*_^*2*^ = 0.63; *R*_*m*_^*2*^ = 0.22), though the annual shift was only 100 m at the population level (Fig. 4). ʻIʻiwi did not exhibit seasonality in minimum elevation (*β*_*sin*_ = 7.21; *P* = 0.09, *β*_*cos*_ = 9.78; *p* = 0.03; *R*_*c*_^*2*^ = 0.37; *R*_*m*_^*2*^ = 0.05). Though we found no evidence of seasonality in daily elevation range (*β*_*sin*_ = − 0.35; *p* = 0.21, *β*_*cos*_ = − 0.37; *p* = 0.22; *R*_*c*_^*2*^ = 0.31; *R*_*m*_^*2*^ = 0.01), daily NSD increased May-Aug. (*β*_*sin*_ = − 0.08; *P* = 0.45, *β*_*cos*_ = − 0.55; *p* < 0.001; *R*_*c*_^*2*^ = 0.75; *R*_*m*_^*2*^ = 0.15) for both species. The peak in daily NSD coincided with the zenith of the commuter group, in which long flights (> 1 km from detection centroids) were strongly oriented to the north-northeast from the Pua ‘Akala tract (3.5 ± 0.3°), and thus largely followed elevational gradients.

## Discussion

Using a suite of movement metrics, we characterized the spatial behavior of two tropical nectarivorous birds across the annual cycle and revealed three distinct movement strategies that can concurrently be employed across the population for each species. ʻApapane and ʻiʻiwi both exhibited sedentary, central place foraging, and large-scale commuting behaviors, and a high degree of intraspecific variability at both the population and individual level. While all three movement strategies co-occurred, the propensity of each changed throughout the annual cycle in concert with nectar phenology and breeding cycles. The flexible movement behaviors found in our tropical study system sharply contrast with the stark seasonal movement patterns found in temperate systems [5, 90], and highlight the value of examining understudied tropical systems to better understand the diversity of movement behaviors within species and across taxa.

The movement strategies identified in this study are all characterized by strong fidelity to a core area (e.g., range residency), but vary in the degree to which birds move to and from this core area. The restricted space use, high residence time and low revisitation rate for the sedentary group are characteristic of territorial behavior, in which patrolling multiple locations is more beneficial than frequent revisits to a few sites [7]. Indeed, ʻiʻiwi and ʻapapane are thought to defend flowering trees and areas around nest sites when breeding [91, 92]. In contrast, birds in the commuter and CPF groups revisited the same locations day after day, exhibiting a pattern common in nectarivorous central place foragers, in which systematic revisits to patchy but renewing nectar sources is more beneficial than random area restricted searches [38, 79]. Although birds in both the CPF and commuter group exhibited central place foraging behavior, their foraging movements occurred over markedly different spatial and temporal scales; the commuters specifically made full day (median ~ 6 h) movements between their roost sites and geographically distinct foraging areas, as is typical of commuting species [15], whereas the CPF group typically made shorter movements (median ~ 0.7 h). Commuting behavior is common in seabirds, waterbirds, wading birds, and owls [13, 14, 16, 93, 94]; however, this study is the first to document commuting behavior in a nectarivorous bird, to the best of our knowledge. Though commuting behavior is not usually observed in small passerines (but see [95]), it is still unknown whether this behavior is uncommon, or just poorly documented due to logistical constraints in tracking.

While we identified three distinct clusters, our results highlight that the movement strategies can also be viewed as a continuum of behaviors. Indeed, commuting can be an extreme case of CPF behavior [13], in which longer distance and/or longer duration foraging flights are driven by food availability, risk of leaving the nest/roost area, and habitat selectivity [96, 97]. Thus, an individual can concurrently exhibit both commuting and CPF behavior, much like individual nectarivores can simultaneously exhibit territoriality and CPF behaviors [38]. For instance, birds in the CPF group occasionally made day-long excursions to foraging grounds, while the commuters occasionally made short trips. These results highlight that the *dominant* behavior exhibited in a cluster may not be the *only* behavior used by individuals in that group. This form of within-cluster variability is notable, as it suggests individual birds can respond to changes in resource availability at different time scales by strategically combining territoriality, CPF, and commuting behaviors in various degrees to maximize resource allocation and fitness.

The seasonal timing of the movement strategies, and the northeastern orientation of the commuter flights are consistent with previous findings from this same study site. In the early 2000s, Guillaumet et al. [44] observed ʻiʻiwi from this same study area making large-scale movements during the non-breeding period for three consecutive years to access super-abundant blooms of ‘ōhi‘a that occurred from May-Aug. at a lower elevation area 10–12 km to the north-northeast of the study area [44, 45]. We also observed the commuters moving to this same area from Apr.-Jul. in all 3 years of our study. Though we were unable to measure ‘ōhi‘a bloom during our study, the consistency between our findings and those of Guillaumet et al. [44] hints at regular annual movements that track landscape-level phenology of ‘ōhi‘a bloom. ‘Ōhi‘a bloom also peaks at our high elevation breeding site in late winter and early spring [44, 45], when the sedentary and CPF strategies were most commonly observed, and may explain why birds exhibited smaller movements during this time period. Indeed, other nectarivores have demonstrated a sedentary strategy to access localized, synchronous, and increased space use and movement to seek out intense asynchronous blooms when local resource density declines [32, 33, 36]. However, it is also possible that localized movements varied between the groups in part, due to heterogeneous resource distribution across the core use areas of different individuals.

Regardless of the factors driving the movement behaviors, our results importantly demonstrate that that individual ʻapapane and ʻiʻiwi can strategically switch among movement strategies to meet shifting environmental and internal conditions. As direct evidence of this, one male ʻapapane captured twice in breeding condition exhibited sedentary behavior in Mar. of 1 year, when ‘ōhi‘a bloom was more likely locally abundant, and CPF from the same area in May-Jun. of another year, during the typical period of lower resource density. Further study could help to elucidate endogenous and environmental factors that drive this intraindividual variability.

The patchy and locally unpredictable nature of nectar resources may also be a strong driver of the forays that birds made outside of the tracking array. Forays are thought to allow animals to learn about landscape-level resource patterns, and prospect for new breeding sites [17, 18]. Since nectarivores have well-developed spatiotemporal cognitive abilities [38, 98], and ‘ōhi‘a are long-lived, ʻapapane and ʻiʻiwi likely become more familiar with local bloom patterns over time through large-scale exploration. Furthermore, since the timing of ‘ōhi‘a blooms can vary annually, exploratory forays outside the tracking area may allow birds to assess conditions across the landscape. In contrast, adults of the closely related, but more dominant ‘akohekohe (*Palmeria dolei*) defend well-defined areas throughout the year, and do not exhibit large exploratory movements akin to the forays seen in our study [99]. This Hawaiian honeycreeper is dominant to ʻiʻiwi where they coexist [100], so the ability to defend high-quality territories and foraging trees may afford a more extreme sedentary behavior in which large exploratory movements are not as advantageous.

Despite making long-distance movements, many ʻapapane and ʻiʻiwi exhibited homing behavior. For instance, we observed 1) range residency, 2) that the five birds tracked over different years occupied the same area from year to year, and 3) that birds returned at night to night to a central area, even in the breeding season. There are several possible explanations for homing behavior during the non-breeding period. Though none of the birds captured Jul.-Dec. showed signs of breeding, they may have initiated nests or engaged in pre-breeding activities such as pair-bond behavior shortly after capture. Alternatively, birds may have used several short-term foraging areas in distinct locations over the non-breeding period, but our tracking duration for any given individual only captured a single homing event. Serial home sites have been observed for migrating hummingbirds [101], juvenile ‘akohekohe [99], and in central place foraging species [102]. Homing behavior in the non-breeding season also supports the long-held theory that small resident populations of both species maintain a presence on the breeding grounds year-round to retain or establish breeding sites [42, 45, 48, 50]. All the ʻapapane and ʻiʻiwi tracked over two separate periods showed fidelity to the same localized area, and two sedentary ʻiʻiwi with no evidence of breeding in a first tracking period occupied the same sites during a second tracking period in which they both were in breeding condition. Homing behavior outside the breeding season is likely adaptive, as familiarity with an area can help an individual locate resources and avoid predators. ʻIʻiwi that initiate nests earlier have greater reproductive success [45], suggesting that early arrival, or continual defense of a breeding territory may be beneficial for reproductive success. Taken together, the advantages of homing behavior outside the breeding season may be an important counter-selective force to nomadism, and thus maintain the diversity of movement strategies observed.

Though nomadism occurs in tropical nectarivores [33], we found only limited evidence of this behavior. Two birds in the commuter group were not range resident, and thus exhibited patterns consistent with nomadism. It is possible that birds engaged in short-term nomadic movements during forays, when they disappeared from our tracking network for 1–22 days. Of the tracked birds, 75% of the CPF group, and 93% of the commuters made at least one foray outside the tracking array. Relatively short-term bouts of nomadism can occur seasonally [21], or irruptively [8]; however, we found no clear seasonal pattern suggesting the latter is more likely in our study species. It is also possible that nomadic behavior was more common than we detected. Some tagged birds were not detected for enough days to be included in this study, due to either tag failure or nomadic movement outside the 40 km^2^ detection range of our tracking network shortly after capture. Alternatively, Hawaiian honeycreepers may exhibit phase nomadism in which animals are nomadic during only one life stage, or partial nomadism, in which only a portion of the population is nomadic [8]. Kuntz [45] found that many female and young ʻiʻiwi apparently departed their breeding areas for most of the post-breeding period, while many adult males remained, providing support for phase and partial nomadism. If the focal species exhibit phase or partial nomadism, we may have underestimated the overall prevalence of nomadic movement, as we primarily captured and tracked adult males. Understanding the prevalence of nomadism in this system requires further study using a wider demographic pool, a larger ARTS network, and aerial flights to survey outside the tracking network.

The movement of Hawaiian forest birds across the landscape has important implications for their risk of exposure to introduced avian malaria. Both the avian malaria parasite and its mosquito vector require sufficient temperatures to develop [52]. Year-round transmission of avian malaria only occurs below ~ 1475 m; however, mosquitoes can develop up to ~ 1715 m m during the warmer weather months (Jul.-Sept.) [53]. The capture locations in our study are above (and just below) the ~ 1715 m disease line, where populations of native birds still occur in high densities [103] with little evidence of disease exposure [104]. However, we recorded birds – especially commuters – making long-distance movements to elevations where avian malaria could be present in the late spring and early summer months, when the mosquito vector occurs at higher elevations. Individuals may have also visited areas with higher avian malaria prevalence during forays outside the tracking array. However, since the *Culex* mosquito vector is primarily nocturnal [48], birds that return to high-elevation core areas at night would have a lower risk of exposure to disease. Commuting behavior may therefore improve fitness in birds from high-elevation breeding populations that need to travel to lower elevations to access foraging resources by minimizing the risk of contracting avian malaria during foraging trips. However, future study is needed to assess the prevalence and fitness consequences of commuting behavior in other high elevation breeding populations.

It is notable that while our study area had a strong elevational gradient most of the documented movement was along a relatively constant elevation for both species. The consistency in elevation is important because travel to low elevations can increase exposure to avian malaria, and lead to population declines, even in disease-free high elevation populations [44]. That we found limited seasonal change in elevation is particularly important for ʻiʻiwi, which are more susceptible to mortality from avian malaria than ʻapapane [105]. Though some studies have documented large elevational movements [44], and long-distance flights of ʻiʻiwi [47, 106], including to low elevations following large storms [107], others have found limited evidence of elevational movements in ʻapapane and ʻiʻiwi [31, 43]. Given that mortality rates from malaria are over 40% in ʻapapane and 90% in ʻiʻiwi [46], movement behavior may be under intense selective pressure to favor strategies that reduce exposure to malaria (i.e., commuting behavior over nomadism). Additional research that assesses the propensity for elevational movements in Hawaiian honeycreepers across multiple high-elevation breeding populations would help to inform the conservation and management of these species.

## Conclusions

This work provides the first quantitative assessments of movement behavior in nectarivorous Hawaiian birds and highlights the power of using automated radio telemetry to gain insights into complex movement behaviors of small-bodied species. The automated tracking system allowed for high-resolution location data both temporally and spatially that is not possible with traditional tracking, particularly in challenging mountainous terrain like Hawaiʻi. Our results demonstrate a suite of flexible and facultative movement strategies that evolved in response to a dynamic tropical environment, and a high degree of intraspecific variability that occurs within both populations and individuals. This work also provides insight into how birds use different movement strategies to balance the benefits of resource tracking with the risks of disease exposure. Although the timing of the movement strategies was consistent with seasonal resource patterns, that individuals from the same location simultaneously employed different movement strategies implies that other endogenous factors may also shape movement behaviors [90]. Further research is needed to tease apart the contribution of endogenous factors, resource distribution, breeding phenology, and other environmental factors that may be driving specific movement behaviors.

## Supplementary Information

**Additional file 1: Figure S1.** Example track from a representative bird in the central place forager (CPF), sedentary and commuter group, with the Hakalau Forest National Wildlife Refuge boundary in black and automated telemetry towers as black points. Data are from automated telemetry of ʻapapane (*Himatione sanguinea*) and ʻiʻiwi (*Drepanis coccinea*) in the Hakalau Forest National Wildlife Refuge from January 2014 to June 2016.

## Data Availability

Upon acceptance for publication the data will be made publicly available [70] on the U.S. Geological Survey ScienceBase Catalog Server.

## Abbreviations

AIC: Akaike information criteria
AKDE: Autocorrelated kernel density estimate
ARTS: Automated radio telemetry system (ARTS)
CPF: Central place forager
CTSP: Continuous-time stochastic process
MCP: Minimum convex polygon
NSD: Net squared displacement
PCA: Principal components analysis
SDD: Standard distance deviation
VHF: Very high frequency

## References

[1] Nathan R, Getz WM, Revilla E, Holyoak M, Kadmon R, Saltz D, Smouse PE (2008). A movement ecology paradigm for unifying organismal movement research. Proc Natl Acad Sci U S A.

[2] Shaw AK (2020). Causes and consequences of individual variation in animal movement. Mov Ecol.

[3] Holyoak M, Casagrandi R, Nathan R, Revilla E, Spiegel O (2008). Trends and missing parts in the study of movement ecology. Proc Natl Acad Sci U S A.

[4] Tingley MW, Monahan WB, Beissinger SR, Moritz C (2009). Birds track their Grinnellian niche through a century of climate change. Proc Natl Acad Sci U S A.

[5] Rappole JH (2013). The avian migrant.

[6] Boyle WA, Conway CJ (2007). Why migrate? A test of the evolutionary precursor hypothesis. Am Nat.

[7] Abrahms B, Seidel DP, Dougherty E, Hazen EL, Bograd SJ, Wilson AM, Weldon McNutt J, Costa DP, Blake S, Brashares JS, Getz WM (2017). Suite of simple metrics reveals common movement syndromes across vertebrate taxa. Mov Ecol.

[8] Teitelbaum CS, Mueller T (2019). Beyond migration: causes and consequences of nomadic animal movements. Trends Ecol Evol.

[9] Mueller T, Olson KA, Dressler G, Leimgruber P, Fuller TK, Nicolson C, Novaro AJ, Bolgeri MJ, Wattles D, DeStefano S, Calabrese JM, Fagan WF (2011). How landscape dynamics link individual- to population-level movement patterns: a multispecies comparison of ungulate relocation data. Glob Ecol Biogeogr.

[10] Franklin DC, Smales IJ, Quin BR, Menkhorst PW (1999). Annual cycle of the helmeted honeyeater *Lichenostomus Melanops Cassidix*, a sedentary inhabitant of a predictable environment. Ibis..

[11] Ylitalo AK, Heikkinen J, Kojola I (2020). Analysis of central place foraging behaviour of wolves using hidden Markov models. Ethology..

[12] Markman S, Pinshow B, Wright J, Kotler BP (2004). Food patch use by parent birds: to gather food for themselves or for their chicks?. J Anim Ecol.

[13] Elliott KH, Woo KJ, Gaston AJ, Benvenuti S, Dall'Antonia L, Davoren GK (2009). Central-place foraging in an arctic seabird provides evidence for Storer-Ashmole’s halo. Auk..

[14] van Gils JA, Spaans B, Dekinga A, Piersma T (2006). Foraging in a tidally structured environment by red knots (*Calidris Canutu*s): ideal, but not free. Ecology..

[15] Masse RJ, Tefft BC, Amador JA, McWilliams SR (2013). Why woodcock commute: testing the foraging-benefit and predation-risk hypotheses. Behav Ecol.

[16] Rogers DI, Piersma T, Hassell CJ (2006). Roost availability may constrain shorebird distribution: exploring the energetic costs of roosting and disturbance around a Tropical Bay. Biol Conserv.

[17] Wheat RE, Lewis SB, Wang YW, Levi T, Wilmers CC (2017). To migrate, stay put, or wander? Varied movement strategies in bald eagles (*Haliaeetus Leucocephalus*). Mov Ecol.

[18] Blakey RV, Siegel RB, Webb EB, Dillingham CP, Johnson M, Kesler DC (2020). Northern goshawk (*Accipiter Gentilis*) home ranges, movements, and forays revealed by Gps-tracking. J Raptor Res.

[19] Austin D, Bowen WD, McMillan JI (2004). Intraspecific variation in movement patterns: modeling individual behaviour in a large marine predator. Oikos..

[20] Schwarzkopf L, Alford RA (2002). Nomadic movement in tropical toads. Oikos..

[21] Lenz J, Bohning-Gaese K, Fiedler W, Mueller T (2015). Nomadism and seasonal range expansion in a large Frugivorous bird. Ecography..

[22] Baylis AMM, Tierney M, Orben RA, de la Pena DG, Brickle P (2020). Non-breeding movements of Gentoo penguins at the Falkland Islands. Ibis..

[23] Singh NJ, Borger L, Dettki H, Bunnefeld N, Ericsson G (2012). From migration to nomadism: movement variability in a northern ungulate across its latitudinal range. Ecol Appl.

[24] Komers PE (1997). Behavioural plasticity in variable environments. Can J Zool.

[25] Culumber ZW, Anaya-Rojas JM, Booker WW, Hooks AF, Lange EC, Pluer B (2019). Widespread biases in ecological and evolutionary studies. Bioscience..

[26] Stutchbury BJM, Morton ES (2001). Behavioral ecology of tropical birds.

[27] Sekercioglu CH (2010). Partial migration in tropical birds: the frontier of movement ecology. J Anim Ecol.

[28] Dingle H (2008). Bird migration in the southern hemisphere: a review comparing continents. Emu..

[29] Franklin DC, Noske RA (1999). Birds and nectar in a monsoonal woodland: correlations at three Spatio-temporal scales. Emu..

[30] Berlin KE, Pratt TK, Simon JC, Kowalsky JR, Hatfield JS (2000). Plant phenology in a cloud Forest on the island of Maui, Hawaii. Biotropica.

[31] Hart PJ, Woodworth BL, Camp RJ, Turner K, McClure K, Goodall K (2011). Temporal variation in bird and resource abundance across an elevational gradient in Hawaii. Auk..

[32] Cespedes LN, Pavan LI, Hazleturst JA, Jankowski JE (2019). The behavior and diet of the shining sunbeam (*Aglaeactis Cupripennis*): a territorial high-elevation hummingbird. Wilson J Ornithol.

[33] Brown ED, Hopkins MJG (1996). How New Guinea rainforest flower resources vary in time and space: implications for Nectarivorous birds. Aust J Ecol.

[34] Boyle WA (2017). Altitudinal bird migration in North America. Auk..

[35] Franklin DC, Menkhorst PW, Robinson JL (1989). Ecology of the regent honeyeater *Xanthomyza-Phrygia*. Emu..

[36] Hazlehurst JA, Karubian JO (2018). Impacts of nectar robbing on the foraging ecology of a territorial hummingbird. Behav Process.

[37] Bergquist CAL (1985). Movements of groups of tui (*Prosthemadera Novaeseelandiae*) in winter and settlement of juvenile tui in summer. N Z J Zool.

[38] Tello-Ramos MC, Hurly TA, Healy SD (2015). Traplining in hummingbirds: flying short-distance sequences among several locations. Behav Ecol.

[39] Jahn AE, Levey DJ, Hostetler JA, Mamani AM (2010). Determinants of partial bird migration in the Amazon Basin. J Anim Ecol.

[40] Mueller-Dombois D, Fosberg FR (1998). Vegetation of the tropical Pacific Islands.

[41] van Dyk KN, Paxton KL, Hart PJ, Paxton EH (2019). Seasonality and prevalence of pollen collected from Hawaiian nectarivorous birds. Pac Sci.

[42] Baldwin PH. Annual cycle, environment and evolution in the Hawaiian honeycreepers (Aves: Drepaniidae). Univ Calif Publ Zool. 1953:285–398.

[43] Ralph CJ, Fancy SG (1994). Demography and movement of the endangered Akepa and Hawaii creeper. Wilson Bull.

[44] Guillaumet A, Kuntz WA, Samuel MD, Paxton EH (2017). Altitudinal migration and the future of an iconic Hawaiian honeycreeper in response to climate change and management. Ecol Monogr.

[45] Kuntz WA (2008). The importance of individual behavior to life history and conservation: breeding and movement biology in the ʻIʻIwi (*Vestiaria coccinea*).

[46] Samuel MD, Woodworth BL, Atkinson CT, Hart PJ, LaPointe DA (2015). Avian malaria in Hawaiian forest birds: infection and population impacts across species and elevations. Ecosphere..

[47] Warner RE (1968). The role of introduced diseases in the extinction of the endemic Hawaiian avifauna. Condor..

[48] Van Riper C, Van Riper SG, Goff ML, Laird M (1986). The epizootiology and ecological significance of malaria in Hawaiian land birds. Ecol Monogr.

[49] Pimm SL, Pimm JW (1982). Resource use, competition, and resource availability in Hawaiian honeycreepers. Ecology..

[50] Ralph CJ, Fancy SG (1995). Demography and movements of Apapane and Iiwi in Hawaii. Condor..

[51] Paxton KL, Kelly JF, Pletchet SM, Paxton EH (2020). Stable isotope analysis of multiple tissues from Hawaiian honeycreepers indicates elevational movement. PLoS One.

[52] LaPointe DA, Goff ML, Atkinson CT (2010). Thermal constraints to the sporogonic development and altitudinal distribution of avian malaria plasmodium relictum in Hawai'i. J Parasitol.

[53] Ahumada JA, Lapointe D, Samuel MD (2004). Modeling the population dynamics of *Culex Quinquefasciatus* (Diptera: Culicidae), along an elevational gradient in Hawaii. J Med Entomol.

[54] US Fish and Wildlife Service (2010). Hakalau Forest national wildlife refuge comprehensive plan.

[55] Hart PJ, Ibanez T, Uehana S, Pang-Ching J (2020). Forest regeneration following ungulate removal in a montane Hawaiian wet forest. Restor Ecol.

[56] Pejchar L, Gallo T, Hooten MB, Daily GC (2018). Predicting effects of large-scale reforestation on native and exotic birds. Divers Distrib.

[57] Paxton EH, McLaughlin R, Levins S, VanderWerf E, Lancaster N (2016). Aging and sexing guide to the forest birds of Hawai’I Island.

[58] Pyle P (1997). Identification guide to north American birds, part I: Columbidae to Ploceidae.

[59] Rappole JH, Tipton AR (1991). New harness design for attachment of radio transmitters to small passerines. J Field Ornithol.

[60] Larkin RP, Raim A, Diehl RH (1996). Performance of a non-rotating direction-finder for automatic radio tracking. J Field Ornithol.

[61] R Core Team (2020). R: a language and environment for statistical computing.

[62] Rowlingson B (2012). Telemetr: radio direction-finding telemetry code. R package version 0.3.

[63] White GC, Garrott RA (1990). Analysis of wildlife radio-tracking data.

[64] McConnell BJ, Chambers C, Fedak MA (1992). Foraging ecology of southern elephant seals in relation to the bathymetry and productivity of the Southern Ocean. Antarct Sci.

[65] Sumner MD, Wotherspoon SJ, Hindell MA (2009). Bayesian estimation of animal movement from archival and satellite tags. PLoS One.

[66] Burnham KP, Anderson DR (2002). Model selection and multimodel inference: a practical information-theoretic approach.

[67] Fleming CH, Fagan WF, Mueller T, Olson KA, Leimgruber P, Calabrese JM (2016). Estimating where and how animals travel: an optimal framework for path reconstruction from autocorrelated tracking data. Ecology..

[68] Fleming CH, Calabrese JM (2020). Ctmm: continuous-time movement modeling.

[69] Calabrese JM, Fleming CH, Gurarie E (2016). Ctmm: an R package for analyzing animal relocation data as a continuous-time stochastic process. Methods Ecol Evol.

[70] Paxton EH, Smetzer JR, Paxton KL (2021). Hawaii Island locations of Apapane and Iiwi from automated radio telemetry tracking system 2014-2016. U.S. Geological Survey Data Release.

[71] Fleming CH, Calabrese JM (2017). A new kernel density estimator for accurate home-range and species-range area estimation. Methods Ecol Evol.

[72] Fleming CH, Fagan WF, Mueller T, Olson KA, Leimgruber P, Calabrese JM (2015). Rigorous home range estimation with movement data: a new autocorrelated kernel density estimator. Ecology..

[73] Winner K, Noonan MJ, Fleming CH, Olson KA, Mueller T, Sheldon D, Calabrese JM (2018). Statistical inference for home range overlap. Methods Ecol Evol.

[74] Fleming CH, Noonan MJ, Medici EP, Calabrese JM (2019). Overcoming the challenge of small effective sample sizes in home-range estimation. Methods Ecol Evol.

[75] Fleming CH, Sheldon D, Fagan WF, Leimgruber P, Mueller T, Nandintsetseg D, Noonan MJ, Olson KA, Setyawan E, Sianipar A, Calabrese JM (2018). Correcting for missing and irregular data in home-range estimation. Ecol Appl.

[76] Nandintsetseg D, Bracis C, Leimgruber P, Kaczensky P, Buuveibaatar B, Lkhagvasuren B, et al. Variability in nomadism: environmental gradients modulate the movement behaviors of Dryland ungulates. Ecosphere. 2019;10(11). 10.1002/ecs2.2924.

[77] Kapota D, Dolev A, Saltz D (2017). Inferring detailed space use from movement paths: a unifying, residence time-based framework. Ecol Evol.

[78] Bracis C, Bildstein KL, Mueller T (2018). Revisitation analysis uncovers spatio-temporal patterns in animal movement data. Ecography..

[79] Berger-Tal O, Bar-David S (2015). Recursive movement patterns: review and synthesis across species. Ecosphere..

[80] Suzuki R, Terada Y, Shimodaira H (2019). Pvclust: hierarchical clustering with p-values via multiscale bootstrap resampling. R package version 2.2–0.

[81] Hennig C (2007). Cluster-wise assessment of cluster stability. Comput Stat Data Anal.

[82] Kaufman L, Rousseeuw PJ (1990). Finding groups in data.

[83] Salvatore M (2020). Rcompanion: functions to support extension education program evaluation. R package version 2.3.25.

[84] Pike N (2010). Using false discovery rates for multiple comparisons in ecology and evolution. Methods Ecol Evol.

[85] O'brien JM, O'brien CS, MCcarthy C, Carpenter TE (2014). Incorporating foray behavior into models estimating contact risk between bighorn sheep and areas occupied by domestic sheep. Wildl Soc Bull.

[86] Geological Survey US (2017). 1/3rd arc-second digital elevation models (DEMs) -USGS National map 3DEP downloadable data collection.

[87] Pinheiro J, Bates D, DebRoy S, Sarkar D, R Core Team (2020). Nlme: linear and nonlinear mixed effects models. R package version 3.1–144.

[88] Hannon ER, Calhoun DM, Chadalawada S, Johnson PTJ (2018). Circadian rhythms of trematode parasites: applying mixed models to test underlying patterns. Parasitology..

[89] Zuur AF, Ieno EN, Walker NJ, Saveliev AA, Smith GM (2009). Mixed effects models and extensions in ecology with R.

[90] Newton I (2008). The migration ecology of birds.

[91] Fancy SG, Ralph CJ, Poole AF, Gill FB (1997). Apapane (*Himatione sanguinea*), version 1.0. Birds of the world.

[92] Fancy SG, Ralph CJ, Poole AF, Gill FB (1998). Iiwi (*Drepanis Coccinea*), version 1.0. Birds of the World.

[93] Gutierrez JS (2014). Living in environments with contrasting salinities: a review of physiological and behavioural responses in waterbirds. Ardeola.

[94] Séchaud R, Schalcher K, Machado AP, Almasi B, Massa C, Safi K, Roulin A (2021). Behaviour-specific habitat selection patterns of breeding barn owls. Mov Ecol.

[95] Howell CA, Dijak WD, Thompson FR (2007). Landscape context and selection for forest edge by breeding brown-headed cowbirds. Landsc Ecol.

[96] Elgin AS, Clark RG, Morrissey CA. Tree swallow selection for wetlands in agricultural landscapes predicted by central-place foraging theory. Condor. 2020;122(4). 10.1093/condor/duaa039.

[97] Lameris TK, Brown JS, Kleyheeg E, Jansen PA, van Langevelde F (2018). Nest defensibility decreases home-range size in central place foragers. Behav Ecol.

[98] Tello-Ramos MC, Hurly AT, Healy SD (2019). From a sequential pattern, temporal adjustments emerge in hummingbird traplining. Integr Zool.

[99] Wang AX, Paxton EH, Mounce HL, Hart PJ (2020). Divergent movement patterns of adult and juvenile ‘Akohekohe, an endangered Hawaiian honeycreeper. J Field Ornithol.

[100] Carothers JH (1986). Behavioral and ecological correlates of interference competition among some Hawaiian Drepanidinae. Auk..

[101] Lopez-Segoviano G, Arenas-Navarro M, Vega E, Arizmendi MD (2018). Hummingbird migration and flowering synchrony in the temperate forests of northwestern Mexico. PeerJ..

[102] Chapman CA, Chapman LJ, McLaughlin RL (1989). Multiple central place foraging by spider monkeys: travel consequences of using many sleeping sites. Oecologia..

[103] Camp RJ, Brinck KW, Gorresen PM, Paxton EH (2016). Evaluating abundance and trends in a Hawaiian avian community using state-space analysis. Bird Conserv Int.

[104] LaPointe D, Gaudioso-Levita JM, Atkinson CT, Egan AN, Hayes K. Changes in the prevalence of avian disease and mosquito vectors at Hakalau Forest National Wildlife Refuge: a 14-year perspective and assessment of future risk: HCSU Technichal Report. Hilo: University of Hawaii Hilo; 2016. p. 1–57.

[105] Atkinson CT, Woods KL, Dusek RJ, Sileo LS, Iko WM (1995). Wildlife disease and conservation in Hawaii: pathogenicity of avian malaria (*Plasmodium Relictum*) in experimentally infected Iiwi (Vestiaria Coccinea). Parasitology..

[106] MacMillen RE, Carpenter FL (1980). Evening roosting flights of the honeycreepers Himatione Sanguinea and Vestiaria Coccinea on Hawaii. Auk..

[107] Perkins RCL (1903). Fauna Hawaiiensis or the zoology of the Sandwich (Hawaiian) isles. vol part 4.

