# Supplementary material for: Individual and seasonal variation in the movement behavior of two tropical nectarivorous birds
Source: Mov Ecol. 2021 Jul 7;9:36. doi: 10.1186/s40462-021-00275-5 (PMC8264974; doi:10.1186/s40462-021-00275-5)
Supplement: Supplementary file 1 — Additional file 1: Figure S1. Example track from a representative bird in the central place forager (CPF), sedentary and commuter group, with the Hakalau Forest National Wildlife Refuge boundary in black and automated telemetry towers as black points. Data are from automated telemetry of ʻapapane (Himatione sanguinea) and ʻiʻiwi (Drepanis coccinea) in the Hakalau Forest National Wildlife Refuge from January 2014 to June 2016. [file 40462_2021_275_MOESM1_ESM.docx]

**Supplementary Information**


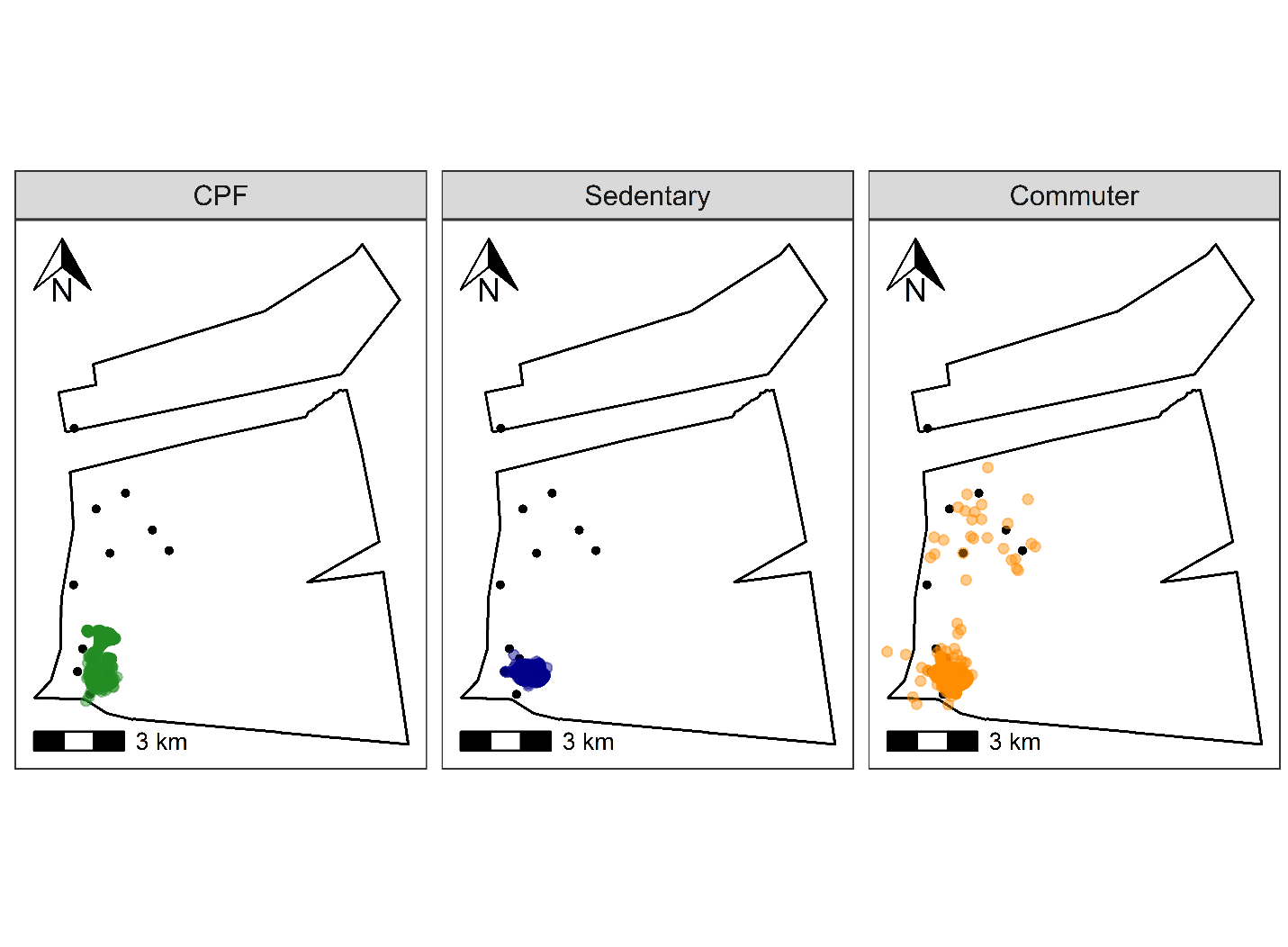


**Figure S1.** Example track from a representative bird in the central place forager (CPF), sedentary and commuter group, with the Hakalau Forest National Wildlife Refuge boundary in black and automated telemetry towers as black points. Data are from automated telemetry of ʻapapane (*Himatione sanguinea*) and ʻiʻiwi (*Drepanis coccinea*) in the Hakalau Forest National Wildlife Refuge from January 2014 to June 2016.
